# Carpal Tunnel Syndrome: Primary Care and Occupational Factors

**DOI:** 10.3389/fmed.2015.00028

**Published:** 2015-05-05

**Authors:** Olivier Saint-Lary, Arnaud Rébois, Zakia Mediouni, Alexis Descatha

**Affiliations:** ^1^Department of Family Medicine, Faculty of Health Sciences Simone Veil, Université de Versailles Saint-Quentin-en-Yvelines, Montigny le Bretonneux, France; ^2^Centre de Recherche en Épidémiologie et Santé des Populations (CESP) – INSERM U1018 Team 1, Villejuif, France; ^3^Medical Home Primary Care of Montcient, Oinville, France; ^4^Occupational Health Unit/EMS (Samu92), Assistance Publique – Hôpitaux de Paris, Poincaré University Hospital, Garches, France; ^5^Population-Based Epidemiologic Cohorts Unit, UMS 011, INSERM, Villejuif, France; ^6^UMS 011 UMR-S 1168, Université de Versailles Saint-Quentin-en-Yvelines, Versailles, France; ^7^VIMA: Aging and Chronic Diseases, Epidemiological and Public Health Approaches, U1168, INSERM, Villejuif, France

**Keywords:** carpal tunnel syndrome, primary care, manual workers, clinical review, pain

## Abstract

Carpal tunnel syndrome (CTS) affects about 1% of working-aged people and is the commonest cause of hand pain in manual workers. CTS is a clinical diagnosis and does not warrant any further investigation in the presence of mild and suggestive CTS. Although the recommended non-surgical management is still a matter of debate, nocturnal splinting or steroid injection are recommended in most countries, with strong to moderate level of evidence for short-term efficacy. Patients with an uncertain diagnosis or severe symptoms, should undergo nerve conduction studies with referral to a hand specialist.

## Introduction

Carpal tunnel syndrome (CTS) affects about 1% of working-aged people and is the most common cause of hand pain in manual workers. It is also a costly musculoskeletal disorder with 450,000 carpal tunnel releases out annually in USA, at a total cost of 2 billion dollars (1). The traditional description of neuropathic symptoms in the median nerve distribution is not the presentation generally observed in primary care (2, 3).

Carpal tunnel syndrome is related to compression and irritation of the median nerve within the carpal tunnel in the wrist: an anatomical space bounded by the carpal bones dorsally and the fibrous flexor retinaculum volarly. Anything that increases the pressure within the compartment or causes a reduction in the volume of this compartment may occasion the symptoms of CTS.

## Usual Clinical Presentation in Primary Care

The possibility of CTS should be considered in the presence of

- paresthesia, dull, aching pain, or discomfort in the hand associated with weakness or clumsiness;
- fluctuating level of symptoms with exacerbation at night (nocturnal numbness), worsened by strenuous hand use or activities with maintained posture (driving);
- and partial relief of symptoms by changing hand posture or shaking the hand.

Patients are rarely able to clearly describe the precise distribution of the symptoms: sensory disturbances from the palm to the fingertips (including the middle finger) in both hands (predominating in the dominant hand in 80% of cases), and radiating to the elbow, are highly suggestive (4).

In contrast, isolated hand pain confined to the ulnar or dorsal aspect of the hand is unlikely to correspond to CTS (5).

## What are the Known Risk Factors?

Known risk factors are pregnancy, diabetes, hypothyroidism, and obesity, in addition to rheumatoid arthritis, renal failure, wrist fracture, and treatment by aromatase inhibitors.

Work exposure is a significant risk factor among manual workers. Blue-collar workers involved in manufacturing, construction, meat- and fish-processing industry, and forestry work with chain saws are most likely to develop CTS, together with lower-grade white-collar women working in personal service industries (6, 7).

Factors of poor prognosis such as fear avoidance behavior, fear of movement, depressive or negative attitude, conflict at home/work, and financial problems should also be searched.

## What You Should Do?

Physical examination includes inspection (color and volume of thenar muscles) and bilateral challenge tests for eliciting symptoms (8, 9):

- Tinel’s test (repetitive tapping on the carpal tunnel six times),
- Phalen’s test (patient wrist in full flexion for 60 s),
- and carpal compression test (firm pressure by the physician on the carpal tunnel for 30 s).

Objective sensory (two-point discrimination) and motor evaluation are important to assess severity of CTS.

Phalen’s sign has a sensitivity ranging from 10 to 73% and a specificity from 55 to 86%.

Tinel’s sign has a sensitivity ranging from 8 to 100% and a specificity from 55 to 87%, the wide ranges probably reflect the difficulty in standardizing the test methods (10).

Both signs are less reliable in advanced CTS.

The following tests should be performed to exclude a differential diagnosis or an associated disorder:

- combined pressure and flexion test at the elbow (firm pressure applied by the physician on the cubital tunnel and full elbow flexion for 30 s) for ulnar nerve entrapment at elbow;
- full extension of patient wrist for 60 s and six repetitive taps on Guyon’s canal (situated on the ulnar aspect of the carpal tunnel between the hook of the hamate bone and the pisiform bone) for ulnar nerve entrapment at wrist;
- resisted wrist flexion or extension that reproduces symptoms of tendinitis;
- pain or paresthesia radiating to the upper extremity with head movements and on active or passive cervical rotation for cervical referred pain or with arm elevation for thoracic outlet syndrome.

## Is Physical Examination Sufficient to Initiate Treatment for CTS?

Carpal tunnel syndrome is a clinical diagnosis and does not warrant any further investigation in the presence of mild and suggestive CTS (3).

Mild and suggestive CTS is defined by

- no severe symptom found
- and no differential diagnosis after physical examination.

Severe symptoms included neurological complications such as thenar weakness, sensory deficit, limitations during work and daily activities are important to assess “functional severity” (writing, opening jars, household chores, carrying grocery bags, bathing, dressing or buttoning clothes, holding the telephone) (11, 12). Severity also includes factors of poor prognosis such as fear of movement, depressive or negative attitude, conflict at home/work, and financial problems.

**Figure 1:**
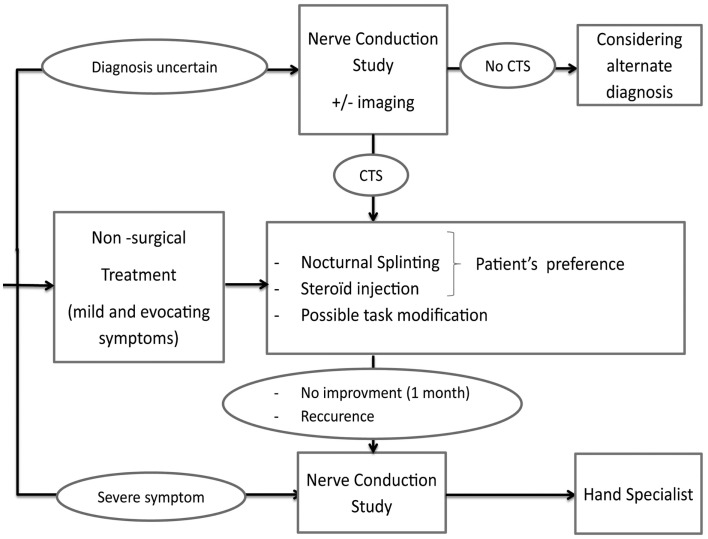
**Treatment algorithm**.

Patients with an uncertain diagnosis or severe symptoms (including factors of poor prognosis) should undergo nerve conduction studies with referral to a hand specialist (Figure 1).

Imaging is not generally performed to establish the diagnosis of CTS, although MRI and X-ray can be useful in the differential diagnosis of hand pain (3).

Ultrasound can detect subtle changes and adds weight to the diagnosis in uncertain cases when the nerve conduction study is normal (13).

## What Treatment could be Proposed in Case of Mild and Suggestive CTS?

Although the recommended non-surgical management is still a matter of debate, nocturnal splinting, or steroid injection are recommended in most countries, with a strong to moderate level of evidence for short-term efficacy (14). The role of oral steroids, non-steroidal anti-inflammatory in local application, ultrasound, yoga, and wrist mobilization remains controversial (15).

### Nocturnal splinting

A removable wrist brace that maintains the wrist at a neutral angle without applying direct compression on the carpal tunnel at night commonly controls the patient’s symptoms (16). Splints can be particularly useful when the patient is repeatedly woken at night by painful paresthesia (2).

The patient must be informed that the method has about 35–80% efficiency in 2–3 weeks and the effects last for 6 months. However, it is relatively inexpensive (<£10 for both hands). The splint should be adjusted to neutral wrist flexion before fitting, because most wrist splints are sold with a moderately extended wrist position to facilitate grasp.

The patient should try different sizes and types of prefabricated splints in a specialized store or pharmacy to determine the most comfortable splint. No serious adverse effects are described. Although daily use is possible, it is not recommended (no benefit, and often too cumbersome for manual workers).

### Steroid injection

**Figure 2:**
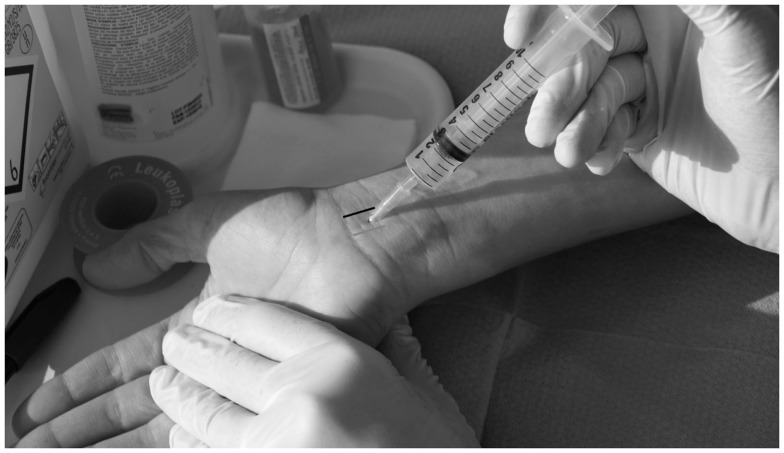
**Steroid injection**. Using a sterile technique, methylprednisolone triamcinolone acetate (longer half-life) with 1 mL of 1% lidocaine is injected using a 25 gage, 1.5″ needle just proximal to the wrist crease, usually between the palmaris longus (gray line) and the flexor carpi ulnaris (black line), between 30° and 45° angle. Disproportionate pain, blood on aspiration or resistance requires immediate adjustment of the needle position (to avoid nerve damage or intravenous/intratendinous injection).

The patient must be informed that steroid injections provide effective temporary relief of symptoms in over 70% of cases 1 month after injection (Figure 2). However, symptoms tend to recur within a few months (for 50–90% of cases depending on the study) (17).

This treatment is particularly effective for pregnant women, as CTS is likely to resolve after delivery (resolution over 80% of cases). In contrast, diabetes is not a good indication for steroid injection (causing elevation of blood glucose and requires closer glycemic monitoring) (18).

Although uncommon (<0.01%), inadvertent steroid injection into the median nerve is a significant hazard leading to temporary or permanent sensory loss, and the practitioner must therefore be familiar with the injection technique (referral needed in that case). Intravenous/intratendinous injections are also theoretically possible.

### Work adaptation

In cases with high-physical exposure, especially for patients working with vibrating tools or manipulating objects with a firm grip, temporary changes in daily work tasks for at least 1 month should be discussed with the workers (2).

For instance, it might be recommended to mix work patterns, increase the number of rest breaks, and if feasible, to change tools (or at least hands), especially vibrating or manipulating objects with a firm grip and awkward wrist posture (>60° of flexion or extension) (7).

Even though computer use is not a risk factor of CTS (19, 20), keyboard or mouse wrist rests for intensive computer workers could be suggested (21).

When these adjustments are not possible, a short sick leave (<2 weeks) is sometimes necessary for workers with intense physical exposure.

## What to do in Case of an Uncertain Diagnosis or Severe CTS?

The patient should undergo nerve conduction studies and be referred to a specialist (Figure 1).

Patients with severe symptoms or those with persistent symptoms after 3 months of conservative treatment should be referred for nerve conduction studies and should be informed about the surgical decompression option.

### Surgical decompression (patient information)

Patients with severe symptoms or those with persistent symptoms after 1 month of conservative treatment should be referred for nerve conduction studies and should be informed about the surgical decompression option (22).

Either open or endoscopic surgery may be used depending on the availability of local expertise and the patient’s preference (although endoscopic release may allow patients to return to work sooner) (23).

Patients should be advised to avoid using a firm grip for 6 weeks.

Surgical failure (5–25% of cases) is mostly attributable to misdiagnosis, delayed treatment to a point where median nerve function is beyond recovery (i.e., the importance of assessing severe symptoms) and complex regional pain syndrome.

A small minority of failures are the result of surgical errors or more unpredictable surgical complications such as infection and painful scarring. No specific recommendations exist about how to manage recurrent or chronic CTS in primary care after surgical failure. After reconsideration, a differential, symptomatic treatment is often necessary, including medications, physiotherapy, and work adaptation.

## Conclusion

Carpal tunnel syndrome is a clinical diagnosis and does not warrant any further investigation in the presence of mild and suggestive CTS. Phalen and Tinel do not add very much, especially in advanced CTS.

Although the recommended non-surgical management is still a matter of debate, nocturnal splinting, or steroid injections are recommended in most countries with good level of evidence. The choice relies mainly on patient preference. The possibility of task modification in manual workers with significant work exposure should also be considered.

## Conflict of Interest Statement

The authors declare that the research was conducted in the absence of any commercial or financial relationships that could be construed as a potential conflict of interest.
